# Evaluation of Gallium-68 prostate-specific membrane antigen, positron emission tomography/computed tomography (GA-68 PSMA PET/CT) in recurrent prostate cancer: a retrospective review of initial clinical experience at Tygerberg Hospital

**DOI:** 10.11604/pamj.2024.48.30.38084

**Published:** 2024-05-30

**Authors:** Bright Awadh Sangiwa, Celeste Burger, Annare Ellmann

**Affiliations:** 1 Department of Nuclear Medicine, Stellenbosch University, Tygerberg Hospital, Cape Town, South Africa

**Keywords:** Prostate cancer, ^68^Ga PSMA PET/CT, Tygerberg Hospital

## Abstract

**Introduction:**

prostate cancer recurrence after definitive therapy for organ-confined disease often manifests as rising prostate-specific antigen (PSA) levels without clinically overt disease. ^68^Gallium prostate-specific membrane antigen, positron emission tomography/computed tomography (^68^GaPSMA PET/CT) imaging plays a major role in the management of recurrent prostate cancer. The purpose of this study was to assess the positivity rate of ^68^Ga PSMA PET/CT scans in cases of prostate cancer recurrence, and to compare the results with existing international literature.

**Methods:**

a retrospective analysis of 177 ^68^Ga PSMA PET/CT scans of patients with biochemically proven disease recurrence was performed. The possible association of a positive PSMA PET/CT with the PSA level and Gleason score were analyzed.

**Results:**

a total of 177 ^68^Ga PSMA PET/CT scans were performed in 163 patients (median age 66 years). Of these, 117 (66%) scans detected the site of disease recurrence. Among patients with PSA 0.2-0.99 ng/ml, 23/49 (47%, p<0.0001) were positive, and 20/35 (57%, p<0.0005) were positive in the group of patients with PSA 1.00-1.99. When PSA values were further categorized into PSA <2 ng/ml and PSA ≥2 ng/ml, detection rates were 49% and 86% respectively (p <0.0001). The scans were positive in 65% of patients with Gleason score of <7, 62% with Gleason score of =7 and 68% with Gleason score >7 (p=0.745).

**Conclusion:**

there was an increase in the detection rate with an increase in the PSA. Gleason score was not a predictor of a positive ^68^Ga PSMA PET/CT scan. ^68^Ga-PSMA PET/CT should be prioritized in patients with biochemical recurrence with PSA levels >0.2 ng/ml.

## Introduction

Prostate cancer, the second most common cancer in males, accounted for more than 7% of all new cases of cancer Worldwide, with an estimated 1.4 million new cases in 2020 [1]. The risk factors include older age (>50 years), ethnicity (higher risk for aggressive disease in black men) and genetic predisposition [2,3]. The definitive diagnosis of prostate cancer is made on prostate tissue biopsy with the histological grading, Gleason score (GS), of importance for prognostic stratification. It defines histological findings into 5 patterns, each describing the degree of differentiation with scores assigned from 1 (most differentiated) to 5 (least differentiated) [4,5]. Patients are grouped into low risk (PSA < 10ng/ml and GS <7 and cT1-2a), intermediate risk (PSA 10-20 ng/ml, or GS=7, or cT2b), and high-risk (PSA >20 ng/ml, or GS >7, or cT2c) [4]. Prostate specific membrane antigen (PSMA) is a non-soluble type 2 integral membrane protein with carboxypeptidase activity, expressed on the apical surface of endothelial cells. PSMA is expressed in normal prostate tissue but is overexpressed in malignant prostate tissue. Overexpression is also associated with androgen deprivation, metastatic disease, and castrate resistant prostate cancer.

These findings clearly show the role of PSMA in the high-risk group [5,6]. Ligands targeting the PSMA protein has been the foundation for the development of PSMA based radiopharmaceuticals in nuclear medicine imaging. It is mainly used for the detection of nodal and distant metastases, or a site of recurrence, but may also have a role in detecting the primary prostate tumour and guiding biopsies [5,7]. Prostate cancer recurrence after definitive therapy for organ-confined disease often manifests as rising prostate-specific antigen (PSA) levels without clinically overt disease. The European Association of Urology (EAU) defines early BCR as rising of the serum PSA level to more than 0.2 ng/ml post prostatectomy or more than 2 ng/ml from nadir post external beam radiation therapy [4,6]. ^68^Ga PSMA PET/CT imaging has become the modality of choice for the detection of disease recurrence at low PSA levels [8,9]. The purpose of this study was to assess the positivity rate of ^68^Ga PSMA PET/CT scans performed at Tygerberg Hospital for cases of recurrent prostate cancer and to determine predictors of ^68^Ga PSMA PET/CT positivity.

## Methods

**Study design:** this study was a retrospective analysis of all patients with biopsy confirmed prostate cancer who had ^68^Ga PSMA PET/CT scans performed at the Nuclear Medicine Division (Western Cape Academic PET/CT centre) at Tygerberg Hospital for suspected disease recurrence, between 1^st^ September 2014 and 31^st^ July 2019.

**Study population:** in the study period, ^68^Ga PSMA PET/CT scans in 346 patients with prostate cancer were performed, we included 163 patients who were referred for presumed disease recurrence. Patients who had ^68^Ga PSMA PET/CT scans performed for other indications including disease staging, treatment response, receptor status prior to Lutetium-177 PSMA therapy or other miscellaneous indications were excluded.

**Data collection:** a standardized data collection form was designed to collect information from patient records and included demographic details (name, date of birth, folder number). Clinical information included date of diagnosis, histology, Gleason score, anatomical imaging results, treatment history, date of PET/CT scan, serum PSA levels including nadir PSA and PSA at time of PET/CT scan (not older than 3 months), PET/CT scan results, and number of PET/CT scans. Patients who had previous external beam radiotherapy or brachytherapy were included in the radiotherapy group.

**Statistical analysis:** the data for all patients referred for suspected biochemical recurrence was entered into a Microsoft Excel spreadsheet. The proportions of positive ^68^Ga PSMA PET/CT scans were calculated and compared within subgroups defined by the Gleason Score (Gleason score <7, equal to 7 and >7), the PSA level (<0.2, 0.2-0.99, 1.00-1.99 and ≥2 ng/ml), and initial treatment received by the patients. The various categories were chosen to replicate the methodology in previous studies [10,11]. Descriptive analysis was performed using the Fisher´s test, comparing the different PSA subgroups with the patient group with PSA ≥2 ng/ml, because of the higher patient number in this group. For the Gleason score group analysis, the chi-square test was used. Statistical analysis was performed using GraphPad Prism (Version 9.00, GraphPad Software Inc., GSL Biotech LLC, California, USA). Descriptive statistics, including the Mann-Whitney U test and Kruskal-Wallis Test for categorical variables, were presented in terms of frequency and percentage. The Student´s t-test was employed for numerical variables that met the assumption of normal distribution. In the multivariate analysis, factors identified through univariate analysis were included in logistic regression to ascertain independent predictors. A significance level of p<0.05 was utilized to determine statistical significance. For patients who had more than one scan, the result of the first scan (chronologically) was used in the analysis according to Gleason score and initial treatment.

**Ethical considerations:** this study had approval of Stellenbosch University Health Research Ethics Committee (HREC ref #: S17/04/079). A waiver of informed consent was granted as this is a retrospective study which involved no risk to the subjects.

## Results

**Treatment modalities:** our study group composed of 177 ^68^Ga PSMA PET/CT studies in 163 patients with a median age of 66 years (range 41-85 years). Twelve patients had more than one scan during the study period, with two patients having had 3 PET/CT studies. All patients were diagnosed with prostate cancer recurrence based on the serum PSA level. The primary treatment modality was known in 162 patients, of whom 106 patients (65%) had prostatectomy and 50 patients (31%) had radiotherapy (brachytherapy or external beam radiotherapy). In one patient the treatment modality was not known. The remaining 6 patients (3%) had other treatment either hormonal therapy/ADT, high frequency ultrasound ablation, or intensity modulated radiotherapy (IMRT). These 6 patients were included in our study as the site of disease recurrence was unknown and they had PSA levels at the time of the scan falling in the same range as the patients who had radical prostatectomy or radiotherapy.

**PSA and Gleason score:** in 96 patient scans (54%) the PSA level was <2ng/ml at the time of referral, while for 12 patient scans (7%) the PSA levels were below 0.2 ng/ml. The PSA range was 0.01-49 ng/ml (median = 1.83ng/ml). The Gleason score was documented in 145 patients; the other patients´ Gleason scores could not be obtained due to unavailability of the initial histopathology results. The number of patient scans per PSA category and number of patients per Gleason score category is shown in Table 1.

**Table 1:** number of ^68^Ga PSMA PET/CT scans per PSA category and number of patients per Gleason score category

PSA levels, ng/ml (n=177)	Total, n (%)
<0.2	12 (7)
0.2-0.99	49 (27)
1.0-1.99	35 (20)
≥2	81 (46)
<2	96 (54)
≥2	81 (46)
**Total**	**177 (100)**
**Gleason score** (n=145)	
<7	26 (18)
=7	68 (47)
>7	51 (35)
**Total**	**145 (100)**

PSA:prostate specific antigen; PSMA: prostate specific membrane antigen

**Figure 1:**
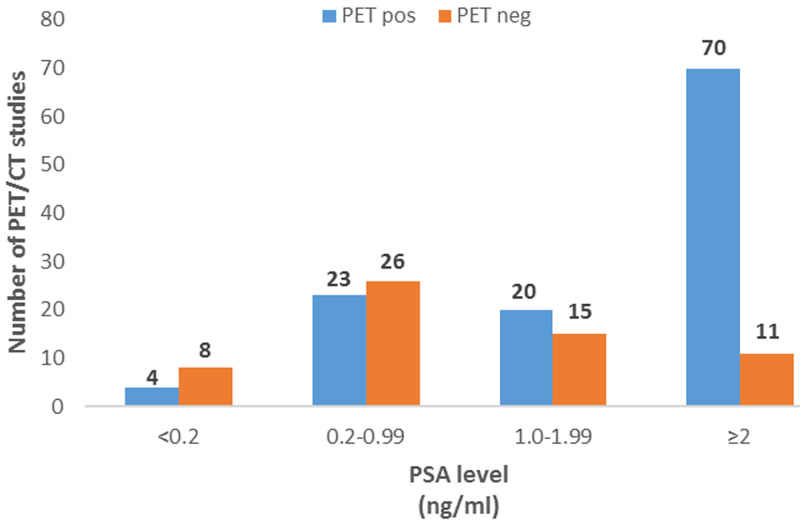
detection rate of ^68^Gallium prostate-specific membrane antigen, positron emission tomography/computed tomography according to prostate-specific antigen (PSA) level

**^68^Ga PSMA PET/CT scan positivity and sites of recurrence:** overall, 66% (n=117) of ^68^Ga PSMA PET/CT scans positively identified a site of recurrence, as tabulated in Table 2. Local disease recurrence limited to the prostate or prostate bed and multiple involved sites were the most reported positive findings. Eighty-two percent (41/50) patients who had radiotherapy as initial treatment had a positive scan compared to 58% (61/106) patients who had prostatectomy (p=0.0027) and 6/6 (100%) of patients who had other treatments e.g., combined androgen blockage (p>0.04). The results of the ^68^Ga PSMA PET/CT scans according to the different PSA categories is shown in Figure 1. The differences between the various PSA subcategories when compared to the PSA category ≥ng/ml were statistically significant. Patients with PSA ≥ ng/ml (n=81) had the highest number of positive scans (70/81, 86%), while only 4/12 (33%) of the scans in the group of patients with PSA <0.2 ng/ml were positive (p<0.0001). In the group of patients with PSA 0.2-0.99 ng/ml, 23/49 (47%) were positive (p<0.0001), and 20/35 (57%) were positive in the group of patients with PSA 1.00-1.99 (p<0.0005). When PSA values were further categorized into PSA <2 ng/ml and PSA ≥ ng/ml, detection rates were 49% and 86% respectively (p<0.0001). There was no statistically significant difference in the number of positive ^68^Ga PSMA PET/CT scans between patients with Gleason scores of <7 (17/26, 65%), GS =7 (42/68, 62%) and >7 (35/51, 68%) (p=0.745).

**Table 2:** ^68^Gallium positive prostate specific membrane antigen prostate specific antigen/computed tomography scans scan positivity and regions involved

Site of recurrence on PSMA PET/CT	Number of patient scans (%)
Local disease only (prostate/prostate bed)	31 (26)
Regional nodal disease only	13 (11)
Local and regional nodal disease	10 (8)
Non-regional nodal disease	17 (15)
Visceral/bone metastases	17 (15)
Multiple sites (including distant/non regional nodal metastases)	29 (25)
**Total number of positive prostate specific membrane antigen prostate specific antigen/computed tomography scans**	**117 (66**)

## Discussion

This retrospective review reports our centre´s clinical experience of using ^68^Ga PSMA PET/CT in patients with recurrent prostate cancer. Most of the available literature on ^68^Ga PSMA PET/CT describe its utility in this group of patients. Many of these studies included patients with low serum PSA levels [12-14]. Our study showed an increase in the detection rate with an increase in the PSA. Gleason score was not a predictor of a positive ^68^Ga PSMA PET/CT scan. The site(s) of recurrence were identified in 66% of patients, with the highest detection rate in patients with PSA ≥2 ng/ml (86%). On the other side of the spectrum, a 33% detection rate was found in patients with a PSA of <0.2 ng/ml. The increase in ^68^Ga PSMA PET/CT scan positivity with an increase in PSA levels has also been described in previous studies [10,12,13,15]. A systematic review by Perera *et al*. [16] reported positive PET/CT scans in 33% of patients with PSA levels of <0.2 ng/ml, compared to 95% in patients with PSA >2 ng/ml. These results compare well with our findings. Although a lower detection rate is seen in the patients with PSA levels below 0.2 ng/ml, this is superior compared to what is reported for conventional imaging modalities. ^68^Ga PSMA PET/CT has a higher sensitivity in detecting sites of recurrence compared to computed tomography (CT) and magnetic resonance imaging (MRI) in patients with low PSA levels [12,16]. A recent study by Radzina *et al*. [17] in patients with early disease recurrence showed ^68^Ga PSMA PET/CT had superior sensitivity (83%) for the detection of local lymph node and bone metastases compared to MRI (42%). Magnetic resonance imaging however, had a higher sensitivity for the detection of local recurrence in the prostate bed (91%) compared to ^68^Ga PSMA PET/CT (64%). Other studies using multiparametric MRI (mpMRI) reported a low positivity of 12-13% in patients with PSA levels <0.5 ng/ml [18,19].

Previously published studies using ^68^Ga PSMA PET/CT also showed a higher sensitivity (83%) compared to bone scan (50%) for the detection of bone metastases [20]. Gomez *et al*. [20] reported that all patients with PSA levels below 7 ng/ml had negative bone scans. This finding could not be tested in our study, as not all patients referred for PET/CT studies had bone scans. The sensitivity of CT is limited for detecting disease recurrence when serum PSA is low. Kane *et al*. [21] reported a low probability of a positive CT scan (14%) in patients with biochemical recurrence after radical prostatectomy. Beresford *et al*. [22] reported a very low positivity rate of 11-14% using CT in patients with even higher PSA levels (mean PSA=12.4 ng/ml). The low sensitivity of CT for nodal metastases is partly due to the lack of CT-based criteria for the detection of metastases in normal-sized lymph nodes. CT is also limited for the detection of skeletal metastases and visceral metastases [23,24]. The study showed no statistically significant association between the Gleason score and ^68^Ga PSMA PET/CT scan positivity. This was also reported by Afshar-Oromieh *et al*. [13] and Perera *et al*. [16].

The study had 156 patients who initially had curative intent therapy with either radiotherapy (50 patients) or radical prostatectomy (106 patients). The proportion of positive scans in the previous radiotherapy group (82%) was higher compared to the previous prostatectomy group (58%, p=0.0027). This could be due to more advanced disease in the radiotherapy group. Meredith *et al*. [25] also reported more positive ^68^Ga PSMA PET/CT scans in patients who had previous radiotherapy (95.3%) compared to patients who had radical prostatectomy (52%). Similarly, Perera *et al*. [16] reported significant differences in scan positivity between patients previously treated with either radical prostatectomy (22%) or radiotherapy (52%). Afaq *et al*. [26] reported local disease (involving the prostate or prostate bed) was most frequently the site of recurrence detected by ^68^Ga PSMA PET/CT, which was also observed in our study. On the other hand, a significant proportion (25%) of our patient studies showed metastases in multiple sites (including distant/non-regional nodal metastases). The detection of disease recurrence by ^68^Ga PSMA PET/CT assists clinicians in decision-making regarding therapy management [26]. The timing for detection of sites of disease recurrence with ^68^Ga PSMA PET/CT is critical for curative or salvage intent therapy. Patients who had radical prostatectomy with disease recurrence confined to the prostatic bed and low serum PSA levels (increasing from nadir) are offered salvage radiotherapy.

The treatment options for patients who had previous radiotherapy with proven local recurrence remain limited, but there is some evidence for the use of salvage high intensity focused ultrasound, salvage cryosurgical ablation and salvage brachytherapy [4,27]. These treatment options are however not available to public sector patients in South Africa. At Tygerberg Hospital, salvage RT is only offered to patients with disease recurrence who had previous prostatectomy and have PSA levels between 0.2 and 1 ng/ml, when ^68^Ga PSMA PET/CT demonstrates no disease outside of the tumour bed and seminal vesicles or when the PET/CT is negative. This is due to the survival benefit in this group of patients [28]. Afaq *et al*. [26] reported that a change in management plan after ^68^Ga PSMA PET/CT was more frequently associated with higher PSA levels (higher than 1 ng/ml). They also observed a change in management more frequently in patients who initially had radiotherapy (50%) compared to those who had radical prostatectomy (34%). In their paper, changes to management included surgery, radiotherapy, hormonal therapy and chemotherapy. This evidence supports the role of ^68^Ga PSMA PET/CT also in patients with higher PSA levels and in those who had previous radiotherapy. This study was limited by its retrospective nature, including the quality of the patient information documented in patient folders. Histopathological confirmation of the identified lesions was not available and therefore the diagnostic accuracy of ^68^Ga PSMA PET/CT scans at our centre could not be accurately determined. Follow-up information about the patients´ further treatment and outcome was also not available. Despite this, our study adds to the limited literature on prostate cancer in the South African population. Our study showed a satisfactory detection rate in patients with PSA levels between 0.2 and 2 ng/ml, and excellent detection rate in patients with PSA > 2 ng/ml. As ^68^Ga PSMA PET/CT is a limited resource in the country, it should be prioritised in patients with biochemical recurrence with PSA levels >0.2 ng/ml.

## Conclusion

The data from our center has demonstrated that ^68^Ga PSMA PET/CT detects the site of recurrence in 66% of patients with recurrent prostate cancer. An increase in the detection rate was observed with an increase in the PSA, with excellent detection in patients with a PSA > 2 ng/ml. Our detection rates at low PSA levels (< 2=ng/ml) are comparable to what is reported at other centers. Gleason score is not a predictor of a positive ^68^Ga PSMA PET/CT scan. Patients who had previous radiotherapy as primary treatment had a higher chance of having a positive ^68^Ga PSMA PET/CT. In our setting, ^68^Ga PSMA PET/CT should be prioritised in patients with biochemical recurrence with PSA levels > 0.2ng/ml.

### What is known about this topic

- *Role of ^68^Gallium prostate-specific membrane antigen, positron emission tomography/computed tomography in biochemical recurrence;*
- *Positivity rate of ^68^Gallium prostate-specific membrane antigen, positron emission tomography/computed tomography in biochemical recurrence in relation to prostate-specific antigen levels*.

### What this study adds

- *First study to report role of ^68^Gallium prostate-specific membrane antigen, positron emission tomography/computed tomography in biochemical recurrence in South Africa and the region, data collected from local population; the study will communicate the experiences and challenges which will accelerate the use of PET/CT modality in our settings;*
- *The study describes initial experience and challenges, especially on missing data for future research in PET; positron emission tomography is gaining a noble recognition in Africa proper collection of patients’ data is important to accelerate future research*.
